# Population genomics and geographical parthenogenesis in Japanese harvestmen (Opiliones, Sclerosomatidae, *Leiobunum*)

**DOI:** 10.1002/ece3.3605

**Published:** 2017-11-23

**Authors:** Mercedes Burns, Marshal Hedin, Nobuo Tsurusaki

**Affiliations:** ^1^ Department of Biology San Diego State University San Diego CA USA; ^2^ Faculty of Agriculture Tottori University Tottori Japan; ^3^ Department of Biological Sciences University of Maryland Baltimore County, Baltimore MD USA

**Keywords:** geographical parthenogenesis, mitonuclear discordance, Opiliones, population genomics

## Abstract

Naturally occurring population variation in reproductive mode presents an opportunity for researchers to test hypotheses regarding the evolution of sex. Asexual reproduction frequently assumes a geographical pattern, in which parthenogenesis‐dominated populations are more broadly dispersed than their sexual conspecifics. We evaluate the geographical distribution of genomic signatures associated with parthenogenesis using nuclear and mitochondrial DNA sequence data from two Japanese harvestman sister taxa, *Leiobunum manubriatum* and *Leiobunum globosum*. Asexual reproduction is putatively facultative in these species, and female‐biased localities are common in habitat margins. Past karyotypic and current cytometric work indicates *L. globosum* is entirely tetraploid, while *L. manubriatum* may be either diploid or tetraploid. We estimated species phylogeny, genetic differentiation, diversity, and mitonuclear discordance in females collected across the species range in order to identify range expansion toward marginal habitat, potential for hybrid origin, and persistence of asexual lineages. Our results point to northward expansion of a tetraploid ancestor of *L. manubriatum* and *L. globosum*, coupled with support for greater male gene flow in southern *L. manubriatum* localities. Specimens from localities in the Tohoku and Hokkaido regions were indistinct, particularly those of *L. globosum*, potentially due to little mitochondrial differentiation or haplotypic variation. Although *L. manubriatum* overlaps with *L. globosum* across its entire range, *L. globosum* was reconstructed as monophyletic with strong support using mtDNA, and marginal support with nuclear loci. Ultimately, we find evidence for continued sexual reproduction in both species and describe opportunities to clarify the rate and mechanism of parthenogenesis.

## 1 INTRODUCTION

### 1.1 Geographical parthenogenesis as a natural laboratory

The evolution of parthenogenesis is a long‐standing biological question inextricably tied to the “paradox of sex” (Burke & Bonduriansky, 2017; de Vienne, Giraud, & Gouyon, 2013), because the vast majority of animal life reproduces sexually (Ashman et al., 2014). Parthenogenesis, asexual reproduction from unfertilized ova, is not uncommon, but is frequently obligate or ontogenetic (Van der Kooi & Schwander, 2015). Truly facultative parthenogenesis in animals has seldom been identified (Van der Kooi & Schwander, 2015), but the novelty of such systems is critical to understanding the widespread nature of obligate sexual reproduction (Tilquin & Kokko, 2016). In order to understand reproductive mode transitions and the costs and benefits of facultative asexuality, species systems that present populations of mixed mode must be identified (Burke & Bonduriansky, 2017). Such within‐population, reproductive polymorphism is possible in species that exhibit geographical parthenogenesis. Geographical parthenogenesis is the particular spatial distribution inhabited by species either wholly or partially reproducing by asexual means (Kearney, 2005; Tilquin & Kokko, 2016) and may naturally provide case studies of the mechanisms underlying the maintenance of sexual reproduction. Parthenogens' distributions are typically broader than those occupied by closely related sexuals (Cosendai & Hörandl, 2010; Verhoeven & Biere, 2013), potentially encompassing harsh and marginal habitats: mountain peaks, deserts, and caves that impose the challenges of extreme temperature and lack of resources on incoming colonists (Kearney, 2005; Mráz, Chrtek, & Šingliarová, 2009).

Understanding the distribution of nonmodel organisms with reproductive polymorphisms therefore has meaningful implications at multiple levels of investigation. The origins and reproductive mode of a species provide critical information to workers concerned with the systematics or conservation of populations (Dejaco, Gassner, Arthofer, Schlick‐Steiner, & Steiner, 2016) or recommending areas for protection. Genomic innovations such as duplication (often the prelude to asexual reproduction; Larkin, Tucci, & Neiman, 2016) may play a role in the construction of complex genomes (Berthelot et al., 2014; Campbell, Ganley, Gabaldón, & Cox, 2016; Glasauer & Neuhauss, 2014; Kellis, Birren, & Lander, 2004; Kenny et al., 2016), thus making these pathways of particular import toward understanding why some lineages are evolutionarily successful and others are not (Pandit, Pocock, & Kunin, 2011). Exploration of the habitats commonly inhabited by parthenogens may also yield predictions about the biotic and abiotic factors that favor transitions in reproductive mode (Tilquin & Kokko, 2016).

A number of hypotheses have been offered as to the mechanisms contributing to geographical parthenogenesis, including selection for the beneficial demographic effects of parthenogenesis (Cosendai, Wagner, Ladinig, Rosche, & Hörandl, 2013; Kearney, 2005), biotic influences (Verhoeven & Biere, 2013), and successive genetic bottlenecks (Haag & Ebert, 2004). Some of these hypotheses are in turn reliant upon the origins and developmental pathways leading to parthenogenesis (Hörandl, 2006): Asexual populations frequently show evidence of hybrid origin (Janko, Kotlík, & Ráb, 2003; Kearney, 2005) and/or polyploidization (Grismer et al., 2014; Larkin et al., 2016; Myers, Trewick, & Morgan‐Richards, 2013), and male‐vectored “contagious” parthenogenesis has also been described (Maccari, Amat, & Gómez, 2013; Tucker, Ackerman, Eads, Xu, & Lynch, 2013; Xu, Innes, Lynch, & Cristescu, 2013). In order to disentangle the underlying processes that lead to the particular distribution of a species, we must consider the environments it occupies and query genetic markers that provide multiple lines of historical information (Barrow, Bigelow, Phillips, & Lemmon, 2015; Jezkova et al., 2015).

An issue that may arise from phylogeographical evaluation of a species with variation in reproductive mode is that the population structure produced via expansion out of refugia may be difficult to distinguish from structure that results from reproductive mode (Wachter et al., 2016). In both cases, populations at high elevation and/or latitude should have less genetic diversity and heterozygosity than their low elevation/southern counterparts, although the mechanism for this would differ (Kearney, 2005; Law & Crespi, 2002). One method for reconstructing the colonization history of marginal habitats seen in geographical parthenogens is to acquire sets of mitochondrial and nuclear markers and evaluate the degree to which both data types associate across a species range (Paczesniak, Jokela, Larkin, & Neiman, 2013). Because mitochondrial and nuclear genomes have different modes of inheritance in animals, they carry patterns of variation that may or may not agree. Mitonuclear concordance may be indicative of a number of speciation mechanisms (Larmuseau, Raeymaekers, Hellemans, Van Houdt, & Volckaert, 2010; Toews & Brelsford, 2012), including hybridization and incomplete lineage sorting (Denton, Kenyon, Greenwald, & Gibbs, 2014; Gompert, Forister, Fordyce, & Nice, 2008; Zakas, Jones, & Wares, 2014). Increasing concordance between the mitochondrial and nuclear genomes may signal co‐adaptation of the genomes (Hadjivasiliou, Pomiankowski, Seymour, & Lane, 2012), but also implicates a decrease in dispersal and/or genetic exchange (Fang, Chen, Jiang, Chen, & Qiao, 2016; Sequeira, Sijapati, Lanteri, & Albelo, 2008; Simon, Delmotte, Rispe, & Crease, 2003), as may be seen in organisms that reproduce facultatively or obligately through parthenogenesis (Paczesniak et al., 2013; Thielsch, Brede, Petrusek, de Meester, & Schwenk, 2009) or other rare fertilization patterns (Eyer, Leniaud, Tinaut, & Aron, 2016; Hedtke & Hillis, 2011; Schaschl, Tobler, Plath, Penn, & Schlupp, 2008).

### 1.2 The Japanese leiobunine harvestmen: model facultative parthenogens?

**Figure 1:**
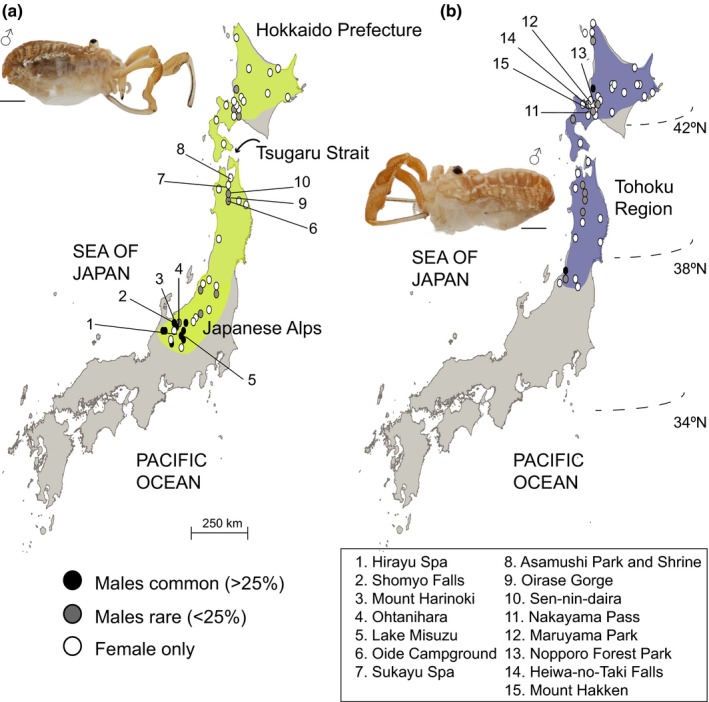
Shaded distribution maps with select geological features and latitude for (a) *Leiobunum manubriatum* and (b) *Leiobunum globosum*, adapted from Tsurusaki (1986). Points indicate survey sex ratio, where white = female only, gray = males rare (<25%), and black = males more common (>25%). Sampling localities 1–15 are plotted across (a) and (b). Photographs of males (legs removed) of each species are inset and accompanied by scale bar of 1 mm

Within the arachnid order Opiliones, also called harvestmen or “daddy‐longlegs,” reports of parthenogenesis are uncommon, although some all‐ or nearly all‐female species are known (Pinto‐da‐Rocha & Giribet, 2007; Wachter et al., 2016). Maternally inherited bacterial endosymbionts that can bias sex ratio and even induce parthenogenetic ability have been described in other arachnids (Baldo, Prendini, Corthals, & Werren, 2007; Duron, Hurst, Hornett, Josling, & Engelstädter, 2008; Goodacre, Martin, Thomas, & Hewitt, 2006; Vanthournout, Swaegers, & Hendrickx, 2011), but these infections have not been shown to render arachnid species parthenogenetically competent. This makes the *Leiobunum curvipalpe* group of Japan particularly unique, as it contains sister taxa *Leiobunum globosum* and *L. manubriatum*, two species that are believed to be facultative thelytokous parthenogens (i.e., unfertilized eggs develop into female offspring). Eggs oviposited by field‐collected virgin females undergo embryogenesis (Tsurusaki, 1986) and the same work identified both diploid and tetraploid races in *L. manubriatum*, while *L. globosum* was hypothesized to be an all tetraploid species. The range of *L. manubriatum* extends from the Japanese Alps region (including Nagano, Toyama, and Gifu Prefectures) of central Honshu Island (Figure 1) to the northernmost island constituting Hokkaido Prefecture. *Leiobunum globosum*, which is known only from the Tohoku region (northern Honshu Island, including Akita and Aomori Prefectures), and Hokkaido (Suzuki & Tsurusaki, 1983; Tsurusaki, 1986), is entirely syntopic with *L. manubriatum*. Both species possess male and female sexes, and while *L. globosum* has a high incidence of gynandromorphism (Tsurusaki, 2001), no evidence suggests the hybrid origin of either *L. manubriatum* or *L. globosum*. Most conspicuously, detailed observations of the sex ratios across populations indicate that male counts decrease with increasing latitude and elevation (Tsurusaki, 1986), supporting geographical parthenogenesis in these species. Secondary sex characteristics used in coercive mating are more pronounced in rare males from female‐biased populations (Burns & Tsurusaki, 2016), suggesting reproductive mode, mating system, or both are influenced by sexual conflict (Burke & Bonduriansky, 2017; Burke, Crean, & Bonduriansky, 2015), wherein selection on male reproductive phenotype is not relaxed (as in Schwander, Crespi, Gries, & Gries, 2013). All evidence therefore points to *L. manubriatum* and *L. globosum* constituting two natural cases for animal reproductive systems that are evidently rare in nature.

Here, we use nuclear SNP and mitochondrial sequence data to compare and contrast the population genetic structure of *L. manubriatum* and *L. globosum* across their respective ranges. Using phylogenetic reconstruction, we assess the potential for hybridization between *L. globosum* and *L. manubriatum*, with the expectation of polyphyly of populations within the regions of species overlap if hybridization is occurring. If *L. globosum* is an asexual lineage of *L. manubriatum*, sympatric *L. globosum* should cluster with *L. manubriatum* in phylogenetic and individual ancestry analyses.

We utilized standard population genetic statistics and an implementation of discriminant analysis of principal components in order to assess direction of range expansion and the distribution of mitonuclear concordance. We anticipated that genetic diversity and heterozygosity would decrease with latitude in both species, but, assuming a pattern of geographical parthenogenesis that mitonuclear concordance would lead to haplogroup classification having greater discriminatory power than locality within species, especially for specimens from Hokkaido, the most northerly region sampled. This is posited under the assumption that the frequency of asexual reproduction would increase with more northerly latitude, and the resultant lack of recombination and inheritance of both mitochondrial and nuclear genomes as a single unit would lead to decreases in genetic variance and increases in mitonuclear concordance, respectively (Simon et al., 2003). We furthermore queried the distribution of ploidy for both species to confirm Tsurusaki (1986) using an alternative method (fluorescence‐associated cell sorting, or FACS, analysis) and to identify potential polyploid allopatry. Our expectation was that polyploids and diploids would not overlap, as genomic incompatibility and sexual conflict both present grounds for the separation of cytotypes (Hörandl, 2006; Kawatsu, 2013; Kearney, 2005).

## 2 MATERIALS AND METHODS

### 2.1 Collection and DNA extraction

We collected forty‐one female *L. globosum* and seventy‐two female *L. manubriatum* from a total of 15 localities across Hokkaido and Honshu islands during July 2014 (Table S1). *Leiobunum globosum* collections covered nine localities corresponding to the Hokkaido and Tohoku regions (Figure 1 locations 6–14: Oide Campground, Sukayu Spa, Asamushi Forest Park and Shrine, Oirase Gorge, Sen‐nin‐daira, Nakayama Pass, Maruyama Park, Nopporo Forest Park, and Heiwa‐no‐Taki Falls), while *L. manubriatum* was collected from fourteen localities, including the Hokkaido, Tohoku, and Japanese Alps regions (Figure 1 locations 1–9, 11–15: Hirayu Spa, Shomyo Falls, Mt. Harinoki, Ohtanihara, Lake Misuzu, Mt. Hakken, and all *L. globosum* localities with the exception of Sen‐nin‐daira). Specimens were collected by hand and identified by abdominal maculation pattern, labrum shape, and association with highly recognizable conspecific males, if present (Suzuki & Tsurusaki, 1983; Tsurusaki, 1985, 1986). Included in the collection were two female and one male *L. tohokuense* samples (Asamushi Forest Park and Shrine), a closely related (Burns & Tsurusaki, 2016) bisexual species, and one female *L. montanum* (Tottori Prefecture: Mt. Hyonosen) to serve as outgroups in phylogenetic analyses. Following identification, samples were immediately preserved in 100% ethanol and ultimately stored at −80°C until extraction, following importation. Genomic DNA was extracted from coxal muscle and leg tissue using the DNeasy Blood and Tissue Kit (Qiagen).

### 2.2 Flow cytometry

Brain or coxal muscle tissue from representatives of *L. globosum* (thirteen female, one male) and *L. manubriatum* (seventeen female, two male) was extracted, chopped with a sterile razor, sieved with 40 μm pipet tip strainers (Bel‐Art Scienceware), suspended in 200 μl of cold 1× phosphate buffered saline, and stained with a 1× propidium iodide and RNAase A solution following manufacturer's protocols (Abcam). Cell samples were incubated on ice in the dark for 30 min, then fluorescence at 488 nm was quantified using a FACSAria cell sorter (BD Biosciences) running FACSDiva software version 6.1.3 as operated by the SDSU Flow Cytometry Core Facility. Fluorescence histograms acquired following gating of subcellular elements in forward‐ and side‐scatter plots were compared with those generated from the two female *L. tohokuense*, as their diploid genomes are expected to be similar in size to *L. globosum* and *L. manubriatum* (Tsurusaki, 1990). Diploid specimens were identified by comparing the fluorescence mean of the highest cell count peak (indicative of cells in G1 phase) to that of *L. tohokuense* females, ±10%. Tetraploid *L. globosum* and *L. manubriatum* individuals were identified where the G1 cell fluorescence was approximately double that of conspecific cell count peaks.

### 2.3 Nuclear genomic and mitochondrial sequencing

Prior to genome digestion, sample genomic DNA was quantified using a Qubit 2.0 fluorometer (Thermo Fisher Scientific) in order to assure a concentration of at least 12.5 ng/μl. To prepare sequence libraries, we followed a customized ddRADseq protocol adapted from Peterson, Weber, Kay, Fisher, and Hoekstra (2012), and Burns et al. (2016). Genomic DNA (500 ng) from each specimen was digested with 100 units each of restriction endonucleases *EcoRI* (5′‐GAATTC‐3′) and *MspI* (5′‐CCGG‐3′) in CutSmart buffer (New England BioLabs) at a reaction volume of 50 μl and for an incubation of 4 hr at 37°C. Enzymes, buffer, and fragments ≤100 base pairs were subsequently removed using 1.5 times the reaction volume of Agencourt AMPure XP (Beckman Coulter) magnetic beads, following manufacturer's protocols. We subsequently estimated purified digest concentrations using the Qubit 2.0 and reordered on a 96‐well plate in order to bring mean concentrations of each of the plate's 12 columns to within 0.5 standard deviations. One sample was diluted to half its digest concentration in order to bring the standard deviation of the column to 1.37.

Eight custom adaptors prepared for the *EcoRI‐MspI* enzyme combination, and with barcode sequences designed for each row of the 96‐well plate, were ligated to genomic fragments using 100 units of T4 Ligase. Samples were incubated at room temperature (23°C) for 40 min, heat killed at 65°C for 10 min, and cooled by 2°C per 90 s for 22 cycles. Samples were pooled by column and purified prior to fragment size‐selection. We used a Pippin Prep (Sage Sciences) automated size‐selection instrument to isolate fragments in a size range of 400–600 bp. Pooled fragments were amplified using the Phusion PCR kit (New England BioLabs) and standard Illumina primers following Burns et al. (2016). After purification, sample molarity was determined using an Agilent Bioanalyzer 2100 (Agilent Technologies) and two equimolar pooled samples were prepared. Resulting libraries were sequenced on two flowcell lanes of an Illumina HiSeq 2500 under the 100‐bp single‐end protocol at the University of California, Riverside, IIGB Genomics Core facility.

Approximately 900 bp of the mitochondrial cytochrome oxidase (CO) genes I and II was isolated and amplified for collected *L. globosum*,* L. manubriatum*, and *L. tohokuense* specimens. Primers and PCR conditions followed Hedin, Tsurusaki, Macías‐Ordóñez, and Shultz (2012). Amplification products were purified using Millipore HTS vacuum plates (EMD Millipore) prior to sequencing. Standard Sanger sequencing in both directions was carried out by Eurofins Genomics (Louisville, KY, USA).

### 2.4 Data analyses

Mitochondrial sequence data were compiled and imported into *Geneious Pro* v. 8.1.7. (http://www.geneious.com; Kearse et al., 2012), where chromatograph files were combined, trimmed, and aligned using *MUSCLE* (Edgar, 2004).

Reduced‐representation genomic sequence data were demultiplexed and processed using the program *PyRAD* v. 3.0.5 (Eaton, 2014). Separate datasets were created, including all samples combined, *L. globosum* plus outgroup taxa, *L. manubriatum* plus outgroup taxa, and *L. globosum* and *L. manubriatum* samples without outgroups for population‐level analyses. For all datasets, only reads with unambiguous barcodes, phred scores ≥20, and loci with one or fewer undetermined bases were retained. Within‐ and between‐sample read clustering were set at a threshold of 95%, and a minimum of 10 reads per locus was required for within‐sample clustering. Up to six shared polymorphic sites per called locus were allowed to accommodate polyploid genomes, but all samples were treated as diploid, thus allowing two haplotypes per polymorphic site. The decision to process all samples as diploids was made because the developmental pathways to tetraploidy may produce tetraploid marker data that are indistinguishable from diploid (Gompert & Mock, 2017) and these developmental pathways are not yet understood in the focal species (Tsurusaki, 1986). For the full dataset, loci were reported only if they were called in at least 50% of samples (56). For the single species sets, we required at least 75% of samples (*L. globosum*: 30; *L. manubriatum*: 54) to have each called locus.

As our assignments of ploidy using FACS were a largely qualitative test, we followed up our diploid and tetraploid identifications using read depth to assess ploidy (Gompert & Mock, 2017). Briefly, this method infers cytotypes by comparing the allelic ratios of heterozygous SNPs identified during variant calling within each individual. We prepared this data by acquiring a *.vcf output file for all specimens from ipyrad (http://ipyrad.readthedocs.io) using the same parameter settings as in the above. Each specimen was represented in this dataset by two columns: Heterozygotic sites were indicated by side‐by‐side read depths for each allele, and homozygotic or missing sites were masked with NAs. The R package *gbs2ploidy* (Gompert & Mock, 2017) was used to estimate cytotype in two ways: (1) without reference to samples of known ploidy and (2) using specimens that had been processed with FACS as a reference set of tetraploids and diploids.

Phylogenetic analyses were carried out using RAxML v. 8.2.4 (Stamatakis, 2014) for maximum likelihood reconstruction and BEAST v. 2 (Bouckaert et al., 2014) for Bayesian reconstruction of *L. globosum*,* L. manubriatum*, and all samples combined plus outgroup taxa. Likelihood trees were constructed with a GTR+Γ model, and 1,000 bootstrap replicates for maximum likelihood. Bayesian analyses employed a log‐normal relaxed clock (Drummond, Ho, Phillips, & Rambaut, 2006) and constant‐population coalescent priors. Two independent runs of 10 million generations and 10% burn‐in were conducted in BEAST, assessing ESS and convergence with Tracer v. 1.6 (Rambaut, Suchard, Xie, & Drummond, 2014). Within species, genetic structure of localities with two or more samples (i.e., omitting singleton *L. globosum* from localities 9 and 14, and *L. manubriatum* from 11) was explored for datasets of both species‐specific and total set loci, using STRUCTURE v. 2.3.4 (Pritchard, Stephens, & Donnelly, 2000). Here, we ran ten replicate analyses of 1,000,000 steps with 10% burn‐in, allowing admixture and setting sample localities as priors. Likelihoods were interpreted by STRUCTURE HARVESTER (Earl & von Holdt, 2012) to identify the number and identity of putative populations using Evanno, Regnaut, & Goudet's Δ*K* criterion (Evanno, Regnaut, & Goudet, 2005).

Arlequin (v. 3.5, Excoffier & Lischer, 2010) was used to calculate population statistics for the mitochondrial dataset, while we employed the R package *hierfstat* (Goudet, 2004) for nuclear data by converting biallelic STRUCTURE data files for both species to “genind” files using the package *adegenet* (Jombart & Ahmed, 2011). We evaluated these genetic profiles across the species regions of the Japanese Alps, Tohoku Region, and Hokkaido Prefecture using five measures: genetic diversity within regions (represented as π, nucleotide diversity, and divided by number of loci for comparison between *L. globosum* and *L. manubriatum* datasets), genetic differentiation among regions (*F*_ST_ and Φ_ST_ plus standard error), inbreeding coefficient among regions (*F*_IS_, plus 95% CI from 1,000 bootstrap replicates), and expected and observed heterozygosity (*H*_e_, *H*_o_). Standard error for π was bootstrapped in R using 5,000 replicates. Heterozygosity was calculated for localities with two or more samples and regressed against latitude with the expectation that more northerly localities would have reduced *H*_o_. Using PopArt v. 1.7 (Leigh & Bryant, 2015), we constructed median‐joining networks (ε = 0; Bandelt, Forster, & Röhl, 1999) for *L. globosum* and *L. manubriatum* mitochondrial DNA.

**Figure 2:**
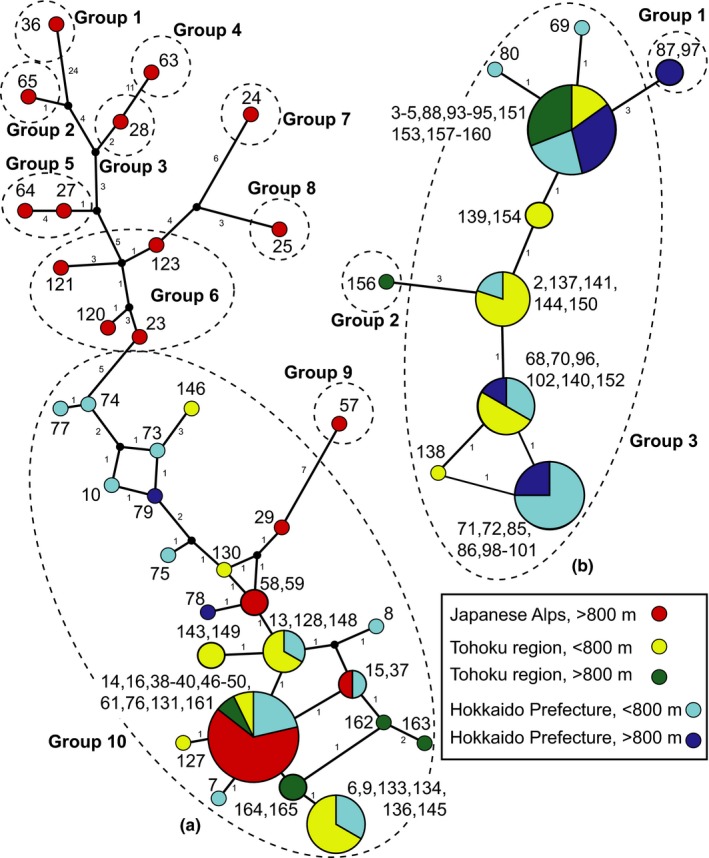
Median‐joining networks (ɛ = 0) of mitochondrial cytochrome oxidase I/II for (a) *L. manubriatum* and (b) *L. globosum*. Small numbers next to branches indicate the number of mutations separating haplotypes. Network haplogroup distinctions are labeled and demarcated with dashed circles. Pie charts indicate the proportion of individuals from each colored locality, with circle size proportionate to the total number of individuals with that haplotype

To evaluate the level of mitonuclear concordance in *L. globosum* and *L. manubriatum*, we used a discriminant analysis of principal components (DAPC; Jombart, Devillard, & Balloux, 2010) to cluster genetically similar individuals by their nuclear SNP profiles with the “dudi.pca” option in the *adegenet* R package. Scale and centering defaults were employed, and missing data were replaced by mean allele frequencies at each given site. Principal components were used to discriminate samples by mtDNA haplogroups for each species using the command “xval.Dapc.” Haplogroups were assigned via two methods: (1) grouping haplotypes to sets separated in the median‐joining mtDNA by three mutational steps for *L. globosum* (1.5% divergence) and 5 mutational steps for *L. manubriatum* (~2% divergence) (Figure 2) to minimize the number of singleton groups and (2) grouping specimens by mtDNA phylogeny, where backbone nodes with 75% or greater support delineated monophyletic groupings (Figure 4). Additional discriminant priors investigated were sampling locality, elevation (above or below 800 m), collection region (Japanese Alps, Tohoku Region, and Hokkaido Prefecture), and a combination of collection region and elevation. As a measure of assignment confidence for each discriminant prior, following Paczesniak et al. (2013), we calculated the maximum posterior group assignment (±95% confidence interval) for each specimen under each discriminant prior. This statistic identifies how well genetic data delineate group membership given the independent geographical or mitochondrially informed priors. Group discrimination confidence means were compared by prior in a Tukey multiple comparisons test. Mean values of maximum posterior group assignment were also contrasted with the probability of correct assignment without genetic information for each species (i.e., each individual has a 1/*N* probability of assignment to any cluster, where *N* = number of clusters for the given prior). This indicates whether nuclear genetic data were better/no better than random group assignment.

## 3 RESULTS

### 3.1 Sequencing, quality control, and haplogroup determination

A total of 158,842,019 reads across all libraries were obtained for nuclear SNP analysis, with an average of 1.13 ± 0.65 million reads per sample following barcode and adaptor trimming. Additional details regarding within‐sample clustering may be found in Table S1. Fastq data files have been deposited to the NCBI Short Read Archive (SRP119937). Two female *L. manubriatum* samples (voucher #s MMB‐30, MMB‐89; NCBI SRA: SRP078623) had been previously sequenced (Burns et al., 2016) and were added to the data analyzed here, yielding 113 total nuclear genomic libraries analyzed. Following variant calls, we recovered 99 SNPs (representing 106 loci) for the *L. manubriatum* dataset, 626 for *L. globosum* samples (representing 626 loci), and 109 (representing 112 loci) for the combined data, including outgroups.

We obtained 901 bp of the COI/II mtDNA region for 106 of the 111 *L. globosum* and *L. manubriatum* specimens collected, and two previously sequenced *L. manubriatum* samples were added to these (voucher # MMB‐30, COI/II accession # KX570871; voucher # MMB‐89, COI/II accession # KX570872). From these data, 31 *L. manubriatum* and 10 *L. globosum* haplotypes were identified. The intraspecific mean divergence (uncorrected *p*‐distance) for *L. globosum* was 0.0108 (±0.0019) and over twice this sum for *L. manubriatum*, at 0.0256 (±0.0124), potentially reflecting the greater overall range size for *L. manubriatum*. Within regions, mean intraspecific divergence decreased considerably on a north–south transect for *L. manubriatum*, with the majority of mtDNA variation found in the Japanese Alps region (0.033 ± 0.013) and the Tohoku (0.011 ± 0.0062) and Hokkaido (0.018 ± 0.0052) regions showing decreased divergence. Little difference in the distribution of mtDNA divergence was seen in *L. globosum* from the Tohoku region (0.0098 ± 0.0026) and Hokkaido Prefecture (0.011 ± 0.0013). After removing sequences with 30% or more missing data, the median‐joining mtDNA network for *L. manubriatum* identified the majority of variation in sequence to be found in the Japanese Alps (Figure 2a). Ten putative haplogroups were defined a posteriori, seven of which constituted of only one sample under our criteria of five mutational steps. For *L. globosum*, the resultant network yielded only three haplogroups separated by three mutational steps, with one singleton from the high elevation Tohoku locality, Sukayu Spa (location 7, Group 2, Figure 2b). The largest *L. globosum* haplogroup, 3, included samples from both the Tohoku Region and Hokkaido Prefecture.

### 3.2 Phylogenetics, population statistics, and cytometrics

**Figure 3:**
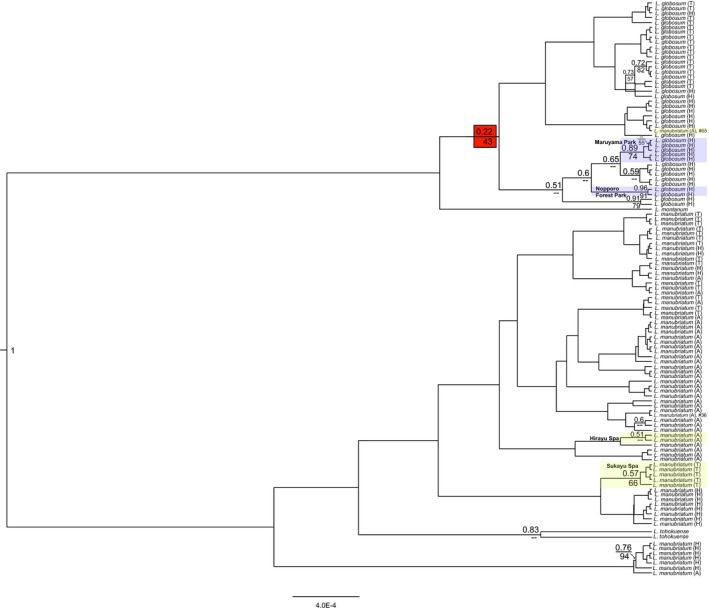
Bayesian likelihood tree reconstructing phylogenetic relationships between all specimens using nuclear SNP data. Posterior probabilities ≥50% are reported to left of node above branches (support for base of *L. globosum* is <50%, and highlighted in red). Bootstrap support ≥50% from maximum likelihood trees is reported to left of node below branches. Supported clades from the same locality are shaded (*L. manubriatum*, yellow, *L. globosum*, blue) and labeled with locality name

**Figure 4:**
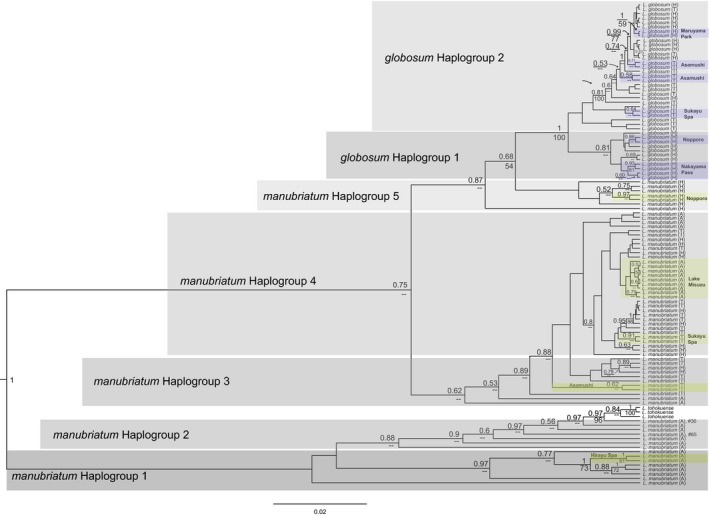
Bayesian likelihood tree reconstructing phylogenetic relationships between all specimens using mtDNA data. Posterior probabilities ≥50% are reported to left of node above branches. Bootstrap support ≥50% from maximum likelihood trees is reported to left of node below branches. Supported clades from the same locality are shaded (*L. manubriatum*, yellow, *L. globosum*, blue) and labeled with locality name. Labeled gray regions denote phylogenetic haplogroups from backbone nodes with greater than 75% support for DAPC prior

Both the maximum likelihood and Bayesian maximum clade credibility trees of the 112 loci nuclear dataset of *all* samples suggested monophyly of *L. globosum* samples (albeit with <50% support) with *L. montanum* as sister; this was not the case for *L. manubriatum* (Figure 3). A cluster of primarily Hokkaido *L. manubriatum* was recovered at the base of *L. manubriatum* plus *L. tohokuense*, and an additional *L. manubriatum* sample from the Japanese Alps (#65, location 3: Mt. Harinoki) was placed within *L. globosum* in the Bayesian tree, although again with little support. Only four sample clusters of three or more specimens at >50% bootstrap support shared localities (shaded boxes, Figure 3; *L. manubriatum*: location 1, Hirayu Spa and 7, Sukayu Spa; *L. globosum*: 12, Maruyama Park). Bayesian and maximum likelihood reconstruction of all specimens using mitochondrial COI/II sequences also found a monophyletic *L. globosum* clade, this time with high support (Figure 4). Paraphyly of *L. manubriatum* is also depicted with the mitochondrial data, with a number of Japanese Alps specimens more closely related to *L. tohokuense* than the remainder of the clade. This analysis recovered a group of Hokkaido Prefecture *L. manubriatum* as sister to all *L. globosum* specimens.

**Figure 5:**
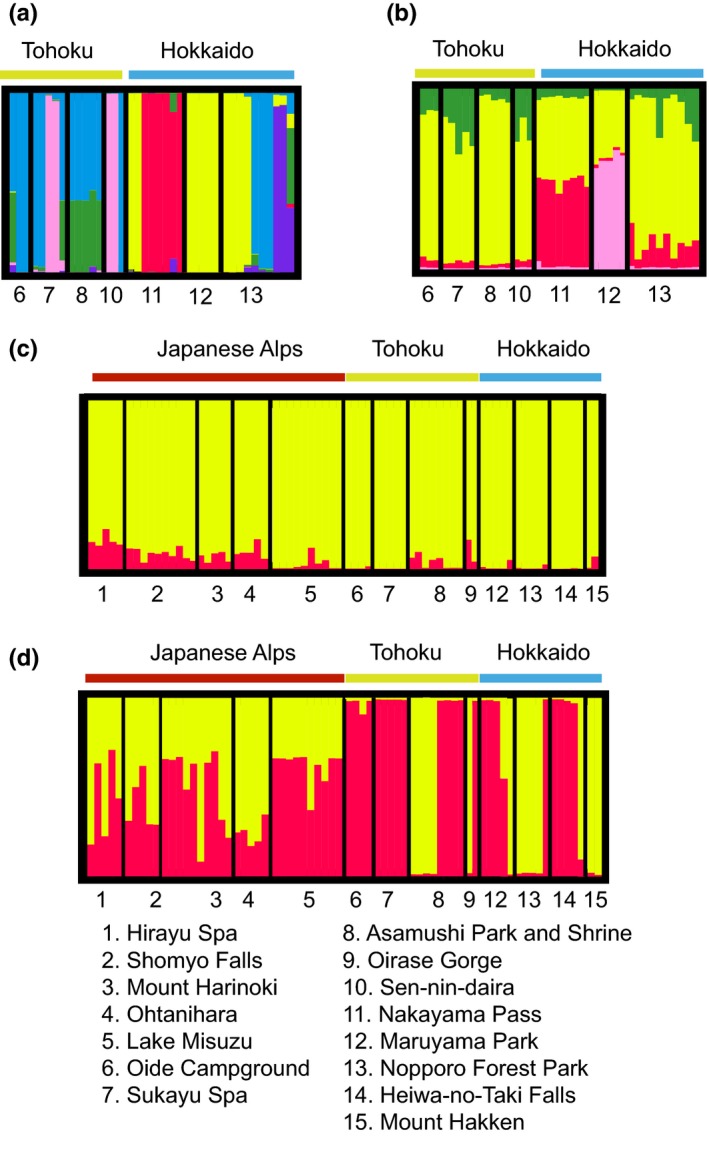
STRUCTURE plots of *L. globosum* (a) *k* = 6 and (b) *k* = 4, the population values supported via the Evanno's *K* method for (a) the species‐specific and (b) 112‐locus total datasets, respectively. (c) and (d) are *k* = 2 STRUCTURE plots for *L. manubriatum*, prepared using either (c) the species‐specific dataset or (d) the 112‐locus dataset common to both species

STRUCTURE analysis using Evanno's Δ*K* method determined best fit population models at *k* = 6 for *L. globosum*, and *k* = 2 for *L. manubriatum* using species‐specific loci, and *k* = 4 for *L. globosum* and *k* = 2 for *L. manubriatum* when limited to the 112 loci with 50% coverage across the dataset. Both analyses identified compositional differences between Tohoku and Hokkaido localities in *L. globosum*, with consistent structure within Tohoku and greater admixture inferred for Hokkaido localities (Figure 5), although the identity of divergent localities within Hokkaido changes based on loci used. For the *k* = 6 model, *L. globosum* from Tohoku and Hokkaido are each inferred members of three populations, although three of ten individuals from location 13, Nopporo Park, are more similar to the Tohoku population signature rather than Hokkaido. In the analysis of the 112 loci dataset supporting *k* = 4, Nopporo Park more closely resembles all Tohoku localities, while the remaining Hokkaido localities (11, Nakayama Pass and 12, Maruyama Park) are divergent. Population assignments at *k* = 2 for *L. manubriatum* outlined greater admixture for Japanese Alps localities as compared to Tohoku and Hokkaido (Figure 5c,d), but there was no differentiation between Tohoku and Hokkaido localities as in *L. globosum*.

**Figure 6:**
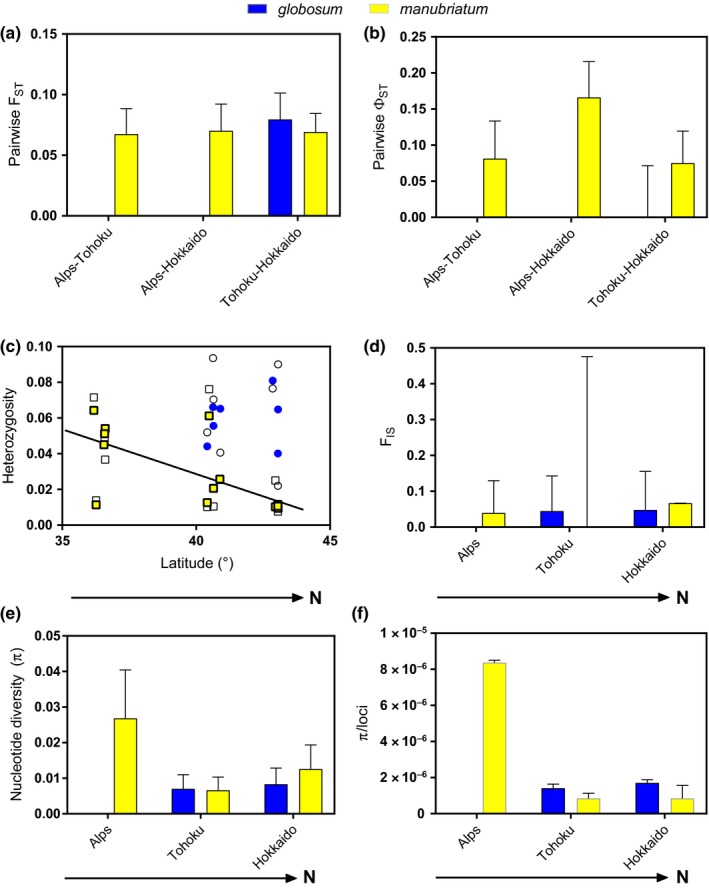
A summary of population statistics, with *L. manubriatum* and *L. globosum* plotted on the same axes. (a) pairwise regional *F*_ST_ using SNP data. (b) Pairwise regional Φ_ST_ with standard error using mtDNA. (c) Observed (*H*_o_, filled symbols) and expected (*H*_e_, open symbols) heterozygosity by locality latitude for *L. globosum* (circles) and *L. manubriatum* (squares). Regression line indicates a significant decrease in *H*_o_ with increase in latitude in *L. manubriatum*. (d) Mean inbreeding coefficient (*F*_IS_) by region, with support values from 1,000 bootstrap replicates. (e) Nuclear data nucleotide diversity (π), normalized by locus count for *L. globosum* and *L. manubriatum*. (f) mtDNA π for *L. globosum* and *L. manubriatum*

We found a significant decrease in both mitochondrial and nuclear genomic nucleotide diversity (π) for *L. manubriatum* when moving from the Japanese Alps to the two northern regions (Figure 6e,f). Although the distinction was less extreme than between the *L. manubriatum* π in the Alps and northern regions, both species also had significantly higher nuclear π in Hokkaido localities as compared to Tohoku, although only *L. manubriatum* mitochondrial π showed this effect (Figure 6e). Observed heterozygosity also decreased with increasing latitude, although the effect was only significant for *L. manubriatum* (Figure 6c; *F*_1,11_ = 8.837, *p* < .05). Considering genetic differentiation, the global *F*_ST_ estimate for *L. manubriatum* was θ = 0.2106 and for *L. globosum* was θ = 0.2784. Pairwise *F*_ST_ values were not different (*F*_3,63_ = 0.1415, *p* = .9347) between the three *L. manubriatum* regions (Table 2; Figure 6a), nor between the two *L. globosum* regions (Table 1). Estimated pairwise values of Φ_ST_ for *L. manubriatum* were highest between samples of the Alps and Hokkaido regions (Figure 6b). Mean inbreeding coefficients calculated in each species across regions of occurrence (Figure 6d) indicated a slight excess (*F*_IS_ = ~0.1) of homozygotes in each region, although confidence estimates for *L. manubriatum* localities in the Tohoku region were particularly wide.

**Table 1:** Pairwise nuclear *F*_ST_ (and 95% CI from 1,000 bootstrap replicates across 626 loci) for *L. globosum* localities for which three or more samples were collected

	Tohoku: 6	Tohoku: 7	Tohoku: 8	Tohoku:10	Hokkaido: 11	Hokkaido: 12	Hokkaido: 13
**Tohoku: 6**	–						
**Tohoku: 7**	0.036679 (0, 0.129)	—					
**Tohoku: 8**	0 (0.341, 0.45)	0 (0.228, 0.34)	—				
**Tohoku: 10**	0 (0.088, 0.225)	0.00062 (0, 0.0599)	0 (0.328, 0.458)	—			
**Hokkaido: 11**	0.192016 (0.313, 0.432)	0.213289 (0.283, 0.398)	0.114193 (0.39, 0.51)	0.17034 (0.298, 0.412)	—		
**Hokkaido: 12**	0 (0.457, 0.619)	0 (0.331, 0.5)	0 (0.522, 0.667)	0 (0.432, 0.524)	0.062289 (0.417, 0.524)	—	
**Hokkaido: 13**	0.063046 (0.44, 0.145)	0.098657 (0.033, 0.116)	0.028464 (0.213, 0.335)	0.07456 (0.076, 0.177)	0.188293 (0.199, 0.298)	0 (0.128, 0.21)	—

Negative values were set to 0.

Thirty‐three *L. globosum* and *L. manubriatum* from 15 localities were examined using flow cytometry to generate fluorescence plots that were compared against those from *L. tohokuense* females. Histograms from two *L. manubriatum* males, both collected in the Japanese Alps region (Shomyo Falls and Mt. Harinoki), closely matched those from *L. tohokuense* females, indicating a diploid genome (Appendix: Fig. S3). Females of *L. manubriatum* had mixed ploidy: Three females, all from the Japanese Alps region (Shomyo Falls, Mt. Harinoki, and Hirayu Spa Area), had diploid genomes, but the additional 14 females surveyed were all tetraploids (Appendix: Fig. S3). Tetraploid female *L. manubriatum* represented samples from all three regions surveyed, including localities in the Japanese Alps (Mt. Harinoki, Lake Misuzu, Ohtanihara) as well as the Tohoku region (Asamushi Shrine, Oide Campground, Sukayu Spa Area, Oirase Gorge) and Hokkaido Prefecture (Heiwa‐no‐Taki Falls, Nakayama Pass, Mt. Hakken, Maruyama Park, Nopporo Forest Park). Of the 14 *L. globosum* surveyed (Tohoku: Asamushi Forest Park, Oide Campground, Sen‐nin‐daira, Oirase Gorge, Sukayu Spa Area; Hokkaido: Nakayama Pass, Nopporo Forest Park), including one male from Nopporo Forest Park, Hokkaido Prefecture, all were estimated to be tetraploid (Table S1).

Using the R package *gbs2ploidy* (Gompert & Mock, 2017), we estimated the probability of cytotype with and without references. Two training datasets were employed—first, using ploidy assignments from FACS analysis, and second, a set that combined FACS data on probable tetraploids with Tsurusaki's, 1986 karyotyping results, which found *L. globosum* to be an entirely tetraploid species. The probability of concurrence between FACS and *gbs2ploidy* was 71%, with 11 of 36 individuals assigned to the opposite cytotype. These mistyped samples included two *L. tohokuense*, although polyploidy in the species has never been described. The probability of correct diploid assignment in *L. manubriatum* was also 71%. As diploid FACS results were only found for *L. manubriatum* from the Alps region, we used a training set that conditioned all *L. globosum* as tetraploids to explore whether refinements to the tetraploid model changed the confidence of diploid assignments. The mean assignment probability to diploidy of Alps *L. manubriatum* species in the test dataset was 43% (min: 0.0016%, max: 100%), but this decreased to 38% (min: 2.9%, max: 97%) when all *L. globosum* were assumed to be tetraploid. Without training data, we estimated the probability of assignment to an unobserved triploid cytotype by allowing 1:2 and 2:1 allelic dosage proportions. The probability of triploidy among our samples was 44%, with the majority (72%) of hypothetical triploids comprised of previously assigned tetraploids.

### 3.3 Discriminant analysis of nuclear principal components

**Figure 7:**
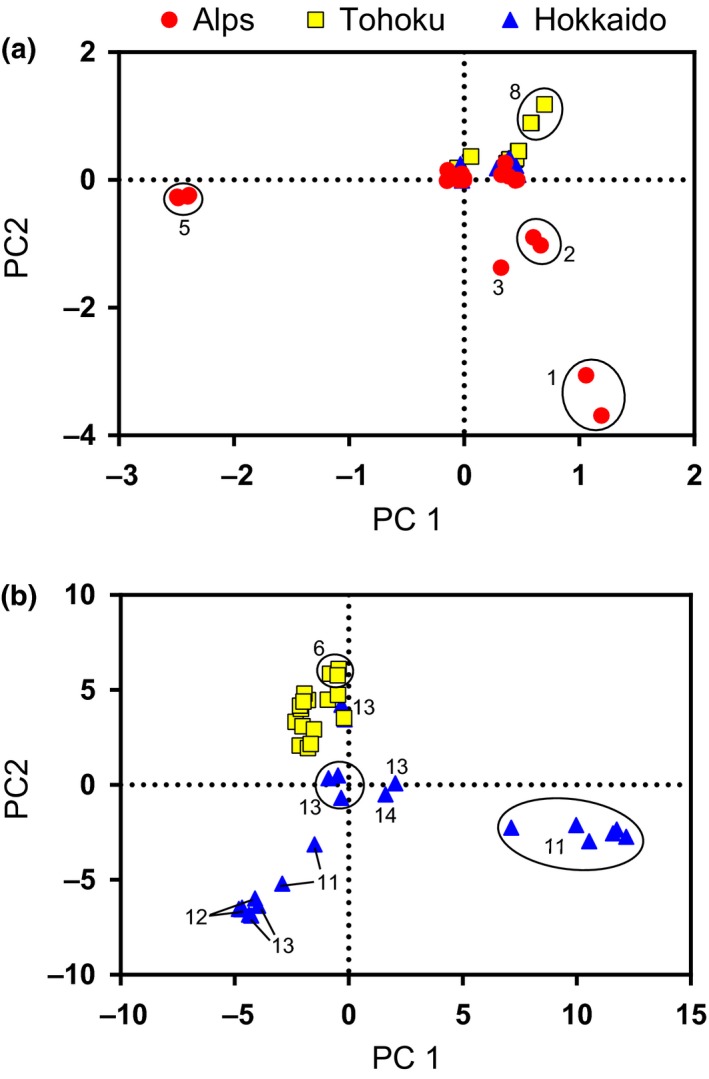
Plot of specimen scores on principal components 1 and 2 for (a) *L. manubriatum* and (b) *L. globosum*, constructed using species‐specific nuclear genomic loci. Regions are indicated by symbol shape and color, where Alps = red circles, Tohoku = yellow squares, Hokkaido = blue triangles. Clusters of specimens from the same locality are circled and selected clusters or individual specimen scores are labeled by localities 1–15 (see Figure 1 for map ID)

We separately analyzed the genomic SNP data acquired for *L. globosum* and *L. manubriatum* using all species‐specific loci, calculating principal components to visualize the phylogeographic structure of the nuclear data (Figure 7). Each set of components was used to discriminate samples using six different assignment priors: locality (nine possible for *L. globosum*, fourteen for *L. manubriatum*), region (two for *L. globosum*, three for *L. manubriatum*), elevation (above or below 800 m), a combination of region and elevation (four for *L. globosum*, five for *L. manubriatum* because Japanese Alps specimens were only found above elevations of 800 m), and mtDNA haplogroup assigned by network (three groups for *L. globosum*, ten for *L. manubriatum*; Figure 2) or phylogeny (two groups for *L. globosum*, five for *L. manubriatum*; Figure 4).

**Figure 8:**
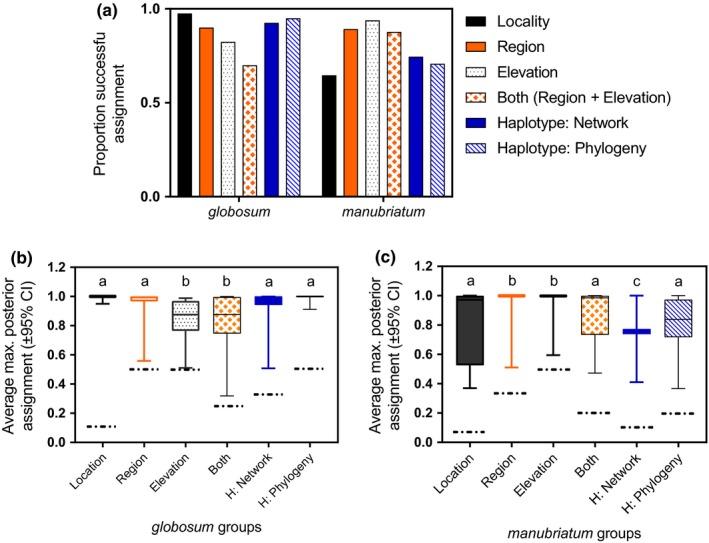
(a) Proportion of successfully assigned individuals in DAPC analyses with six grouping priors (locality, region, elevation, both—a combination of region and elevation, haplotype assigned from median‐joining network, and haplotype assigned by mtDNA phylogeny) for *L. manubriatum* and *L. globosum*. Average maximum posterior probability of assignment (±95% CI) for discrimination of (b) *L. globosum* samples and (c) *L. manubriatum* samples. Letters indicate significant differences in prior confidence following ANOVA with multiple comparisons. Dotted lines indicate mean posterior probability given no genetic principal component data, that is, each individual has a 1/*N* probability of assignment to any cluster, where *N* = number of clusters for the given prior

Plotting specimen scores on the first two principal component axes, we found the region with the greatest divergence for *L. manubriatum* was the Japanese Alps (Figure 7a) and for *L. globosum*, Hokkaido Prefecture (Figure 7b). Comparing the proportion of samples discriminated to the correct group given the prior (Figure 8), we found locality and phylogenetic haplotype to be the best priors for *L. globosum*, with locality achieving the highest maxima of assignment confidence (Figure 8b). Haplogroup was a less informative prior for *L. manubriatum*, as both grouping methods plus locality yielded the lowest proportion of correctly assigned *L. manubriatum* samples based on nuclear SNP data. The highest confidence in *L. manubriatum* DAPC analysis was yielded by the region and elevation priors, with most samples assigned at a posterior probability of essentially 100% (Figure 8c). This is significant, because of the two species investigated, only *L. manubriatum* occurs in the Japanese Alps, where it exclusively occupies habitat at greater than 800 m.

## 4 DISCUSSION

### 4.1 Paraphyly of *Leiobunum manubriatum*—where did *Leiobunum globosum* come from?

Prior to our analyses, many molecular ecologists may have predicted the lack of monophyly of both *L. manubriatum* and *L. globosum*, because the sympatric distribution of the two species (Figure 1) mirrored that of geographically parthenogenetic species, that is, defined by the range expansions of asexual, hybrid, and/or polyploid lineages (Grismer et al., 2014; Janko et al., 2003; Kearney, 2005; Myers et al., 2013; Paczesniak et al., 2013). Northern sympatry might support a hypothesis of *L. globosum* as the species descendant of northern range expansion by thelytokous *L. manubriatum*. Indeed, our cytometric work supports observations made by Tsurusaki in 1986, indicating that all *L. globosum* tested, both male and female, were tetraploid, while *L. manubriatum* had both diploid males and females in the Japanese Alps region, but apparently only tetraploid females in the northern Tohoku and Hokkaido regions. However, we have found support for the monophyly of the all tetraploid *L. globosum* as sister to the Japanese Alps *L. manubriatum*. Furthermore, *L. globosum* males persist (Burns & Tsurusaki, 2016) despite their rarity, further indicating *L. globosum* is neither an all‐female lineage, nor a hybrid of *L. manubriatum* and another *curvipalpe*‐group species. The base of both nuclear and mitochondrial phylogenies is populated by diploid *L. tohokuense* and *L. manubriatum* from apparently diploid populations in the Japanese Alps (Figures 3, 4 and S2). This would suggest that *L. globosum* descended from a common ancestor of both species in the Japanese Alps—most parsimoniously, a tetraploid ancestor.

Even so, like *L. globosum*, all *L. manubriatum* females in the Tohoku and Hokkaido region are tetraploid, indicating homoploid hybridization is at least a possibility (Harrison & Larson, 2014; Yakimowski & Rieseberg, 2014). In fact, when revisiting the Sukayu Spa locality #7 the year following field collection for this work, two of us observed mating between an *L. globosum* male and a female later determined to be *L. manubriatum*. While maintaining the monophyly of *L. globosum*, our phylogenetic results suggest the possibility of mitochondrial introgression from *L. manubriatum* to *L. globosum*, as *L. globosum* nuclear SNPs were more closely related to southern *L. manubriatum*, but *L. globosum* mtDNA was more closely associated with northern *L. manubriatum* (Figures 3 and 4). Additionally, *L. manubriatum* males are particularly uncommon in the Tohoku and Hokkaido regions (Tsurusaki, 1986), suggesting *L. manubriatum* females may be as likely to mate with rare *L. globosum* males as with their own rare conspecifics. Reproductive molecular and behavioral data in these species would be valuable in order to determine the fate of these hybrid crosses, as well as whether northern *L. manubriatum* females resist precopulatory advances of conspecific males from the Japanese Alps (Burns & Tsurusaki, 2016) or male *L. globosum* from their own range.

While analysis of heterozygotic sequence depth in *gbs2ploidy* found some support for triploids in both species, this data had only a moderate rate of agreement (71%) with ploidy estimates from FACS cytometric analysis, and the majority of supposed triploids were classified as tetraploid in the unsupervised analysis. A similar result was found in analysis of simulated 2×/3×/4× populations under low genomic coverage conditions, with the *gbs2ploidy* developers finding that allelic proportions for simulated triploids and tetraploids did not meet theoretical expectations (Gompert & Mock, 2017). The authors hypothesized that the resolution of allelic proportions at higher ploidy levels was especially limited by low sequencing coverage (8× or less). In the present study, our cytological findings suggest a sequencing approach yielding greater coverage (whether by reducing multiplex size or increasing sequencing cycle), combined with technical replication, will be necessary to overcome the challenges associated with sequencing across a duplicated genome.

Consistent with Tsurusaki (1986), we found evidence for diploid and tetraploid *L. manubriatum* females coexisting in the same locality (Alps: Mt. Harinoki #3) as diploid *L. manubriatum* males (Appendix: Fig. S3). It is unclear if this mixed population is representative of two separate races, if tetraploid females behaviorally avoid diploid males (as proposed in theory by Gerber and Kokko (2016) and Kawatsu (2013)), or what the resultant ploidy of putative offspring produced between diploid males and tetraploid females might be. The lack of verified triploid lineages in both *L. manubriatum* and *L. globosum* would suggest that if such matings are productive, some mechanism for the prevention of meiotic reduction in diploid gametes must exist. Ultimately, continued use of cytometric analyses such as FACS or microsatellite development will likely be necessary for successful genotyping, especially among *L. manubriatum* samples from the Japanese Alps, as tetraploids in both species may result from somatic doubling or nondisjunction during meiosis II, leading to allelic dosages identical to diploids (Gompert & Mock, 2017).

### 4.2 Evidence for northward expansion of *L. manubriatum* and *L. globosum*: sexual reproduction continues

As is the case for many geographically parthenogenetic taxa (Hörandl, 2006; Law & Crespi, 2002; Verhoeven & Biere, 2013), our data suggest expansion of the range of *L. manubriatum* proceeded from south to north, with decreasing heterozygosity and nucleotide diversity with increasing latitude (Figure 6). These results are, as per usual in parthenogens, confounded by sex ratio: *L. manubriatum* male numbers are concentrated in the Japanese Alps (Figure 1), where some populations appear to reach equal proportions of observed males and females (Tsurusaki, 1986). Our data may just as easily show evidence of increased sexual reproduction in *L. manubriatum*'s southern range. Certainly, the propensity for male gene flow within this species is greater than that of *L. globosum*, wherein males nearly never reach equal observed ratios with females.

While *F*_IS_ was positive in all regions for both species (Figure 6d), indicating a deficiency of heterozygotes consistent with asexual reproduction, we saw few distinguishing features between localities sampled in the Tohoku region and Hokkaido Prefecture. Hokkaido localities had greater nucleotide diversity than Tohoku localities, but lower observed heterozygosity. Pairwise *F*_ST_ values between localities of these regions do not exceed the *F*_ST_ values between the Japanese Alps and these regions (Tables 2 and 3), despite the greater longitudinal span between the latter. In fact, both *L. globosum* STRUCTURE and DAPC results point to *greater* admixture in Hokkaido localities compared to Tohoku, even when considering only loci found in both species (Figure 5b). We also found little clustering within Tohoku and Hokkaido regions with respect to mtDNA haplotype (Figure 2). This may be due to the geological age and activity of the combined regions. Unlike continental nations, the islands of Japan experienced major crustal movements well into the Quaternary. While the separation between the Honshu and Hokkaido Islands, forming the Tsugaru Strait (Figure 1), is dated to the Pliocene (2.6–5.3 Ma; Uozumi, 1967), tectonic shifts and volcanism in the Oshima Peninsula (Kuno, 1962) may have mediated successive events of separation and reconnection between Hokkaido and Tohoku (Sasa & Izaki, 1961; Uozumi, 1967). While the Tsugaru Strait forms a well‐known biogeographic boundary for vertebrates (the Blakiston Line; Ohsawa et al., 1998), it is possible that this split is less informative for arthropods like harvestmen.

**Table 2:** Pairwise nuclear *F*_ST_ (and 95% CI from 1,000 bootstrap replicates across 106 loci) for *L. manubriatum* localities for which three or more samples were collected

	Alps: 1	Alps: 2	Alps: 3	Alps: 4	Alps: 5	Tohoku: 6	Tohoku: 7	Tohoku: 8	Hokkaido: 12	Hokkaido: 13	Hokkaido: 14
**Alps: 1**	—										
**Alps: 2**	0 (0.011, 0.432)	—									
**Alps: 3**	0.137857 (0.097, 0.364)	0 (0, 0.457)	—								
**Alps: 4**	0 (0.144, 0.532)	0 (0, 103, 0.536	0 (0.002, 0.240)	—							
**Alps: 5**	0.296813 (0.246, 0.686)	0 (0.113, 0.704)	0.10676 (0.059, 0.497)	0 (0.152, 0.590)	—						
**Tohoku: 6**	0.205138 (0.129, 0.568)	0 (0.062, 0.64)	0 (0.004, 0.136)	0 (0.073, 0.386)	0.241058 (0, 0.624)	—					
**Tohoku: 7**	0 (0.212, 0.606)	0 (0.176, 0.669)	0 (0.012, 0.266)	0 (0.195, 0.465)	0 (0, 0.67)	0 (0.01, 0.474)	—				
**Tohoku: 8**	0.17999 (0.162, 0.592)	0 (0.052, 0.597)	0.019349 (0.071, 0.127)	0 (0.06, 0.354)	0.249399 (0.048, 0.567)	0.058745 (0, 0.077)	0 (0.004, 0.388)	—			
**Hokkaido: 12**	0.231321 (0.114, 0.574),	0 (0.034, 0.622)	0.002493 (0, 0.067)	0 (0.064, 0.381)	0.244822 (0, 0.62)	0.113944 (0, 0.25)	0 (0, 0.431)	0.099952 (0, 0.056)	—		
**Hokkaido: 13**	0.201515 (0.116, 0.604)	0 (0.013, 0.637)	0 (0.006, 0.079)	0 (0.022, 0.379)	0.224179 (0, 0.615)	0.121002 (0.042, 0.268)	0 (0, 0.463)	0.053063 (0.025, 0.095)	0.082045 (0, 0.099)	—	
**Hokkaido: 14**	0.250699 (0.145, 0.596)	0 (0.109, 0.678)	0.015706 (0, 0.151)	0 (0.114, 0.41)	0.264544 (0, 0.661)	0.178441 (0, 0.35)	0 (0.032, 0.474)	0.139431 (0, 0.229)	0.077946 (0.012, 0.118)	0.063754 (0, 0.167)	–

Negative values were set to 0.

That we found greater support for mitonuclear concordance in *L. globosum* than in *L. manubriatum* seems to stem in part from the entirely Alps‐derived network haplogroups 1–9 and also from a lack of mtDNA diversity seen in *L. globosum*. It is notable that every individual from network haplogroups 1–9 was either confirmed to be diploid (Table S1) or was collected from a locality where diploids were identified. The majority of Alps‐derived *L. manubriatum* specimens that cluster into the large network haplogroup 10 were all from the same locality, Lake Misuzu (#5), although specimens from Mt. Harinoki (#3) and Ohtanihara (#4) were also represented. All Tohoku and Hokkaido samples clustered into haplogroup 10, suggesting Lake Misuzu may form part of the southernmost boundary of a prevalent haplotype, and potentially, part of the 2×–4× transition zone within the species (Table S1; Appendix: Fig. S3). Northward conservation of haplotypes found in haplogroup 10 may also indicate a role for mitonuclear co‐adaptation in the inclusive populations (Hadjivasiliou et al., 2012; Hill, 2015).

Within species, haplogroup distinction was much less clear cut than was demonstrated in other works (Paczesniak et al., 2013) where there were marked differences in mtDNA that correlated strongly with reproductive mode, and haplogroup number affects DAPC classification statistics (Figure 8). Singleton groups, as were common for *L. manubriatum*, cannot be used in both testing and training datasets for discriminant analyses, reducing the power of the nuclear SNP data to identify true population structure. That said, STRUCTURE and DAPC results lead us to suspect that localities #1–4, which comprise network haplogroups 1–9, are sources of sexual reproduction in *L. manubriatum*. Efforts are underway to evaluate and compare rates of sexual reproduction versus parthenogenesis using SNP genotyping of mothers and offspring from both the Alps and Tohoku regions. Additionally, we might consider the fit of pseudogamous scenarios, as in gynogenetic species (Choleva et al., 2014), where male stimulation of embryogenesis is necessary in order to catalyze reproductive processes (Schlupp, 2005). It is hypothesized that this form of asexuality also develops from rare sexual reproduction (Mikulíček, Kautman, Demovič, & Janko, 2014), and while Tsurusaki disproved the necessity of males for parthenogenesis in *L. manubriatum* (Tsurusaki, 1986), the possibility remains for variation in reproductive mode in isolated populations of the species. Such reproductive mechanisms have never been described in Opiliones, but could explain a portion of the discordance between the nuclear and mtDNA data in these species. The *L. manubriatum* geographical distribution may reflect a gradient of less common reproductive modes such as these, inviting focused research on the reproductive and developmental pathways utilized by the species across its range.

## CONFLICT OF INTEREST

None declared.

## AUTHOR CONTRIBUTIONS

MB, MH, and NT designed the research; MB performed the research; MB, MH, and NT contributed to the reagents/analytical tools MB analyzed the data; MB, MH, and NT wrote the manuscript.

## DATA ACCESSIBILITY

*DNA sequences*

GenBank accession numbers: (KX570871, KX570872, MG201404‐MG201509). NCBI SRA: (Samples MMB_30, MMB_89: NCBI SRA SRP078623; Samples MMB_17, MMB_20, MMB_67: SRA SRS1268470; Remaining specimens: SRA SRP119937).

BEAST *.xml files and R scripts for DAPC, ploidy analyses, and statistics

Data available from the Dryad Digital Repository: https://doi.org/doi:10.5061/dryad.n8147.
