# Supplementary material for: Population genomics and geographical parthenogenesis in Japanese harvestmen (Opiliones, Sclerosomatidae, Leiobunum)
Source: Ecol Evol. 2017 Nov 23;8(1):36–52. doi: 10.1002/ece3.3605 (PMC5756897; doi:10.1002/ece3.3605)
Supplement: Supplementary file 1 [file ECE3-8-36-s001.pdf]

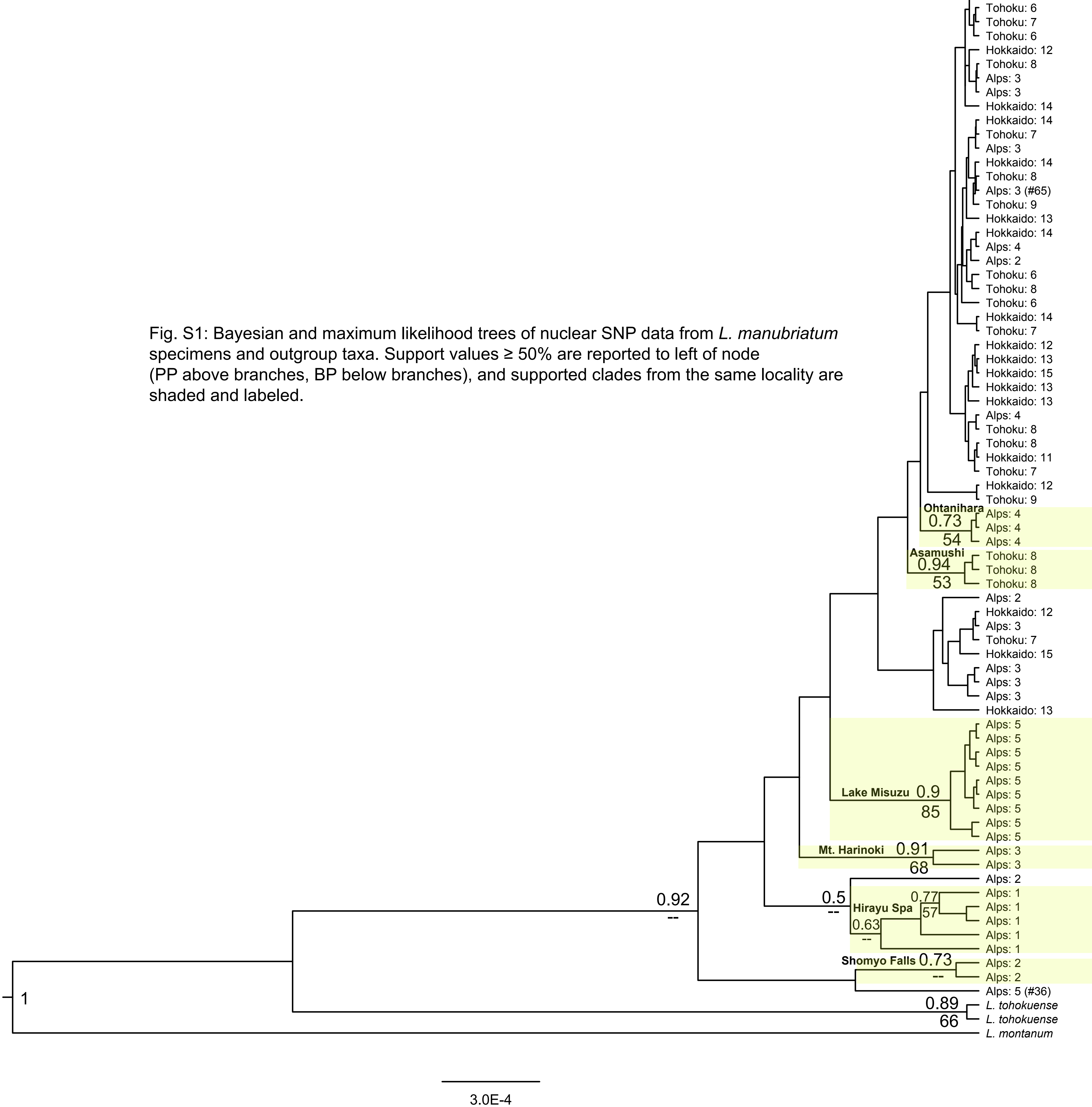

Fig. S1: Bayesian and maximum likelihood trees of nuclear SNP data from *L. manubriatum* specimens and outgroup taxa. Support values ≥ 50% are reported to left of node (PP above branches, BP below branches), and supported clades from the same locality are shaded and labeled.

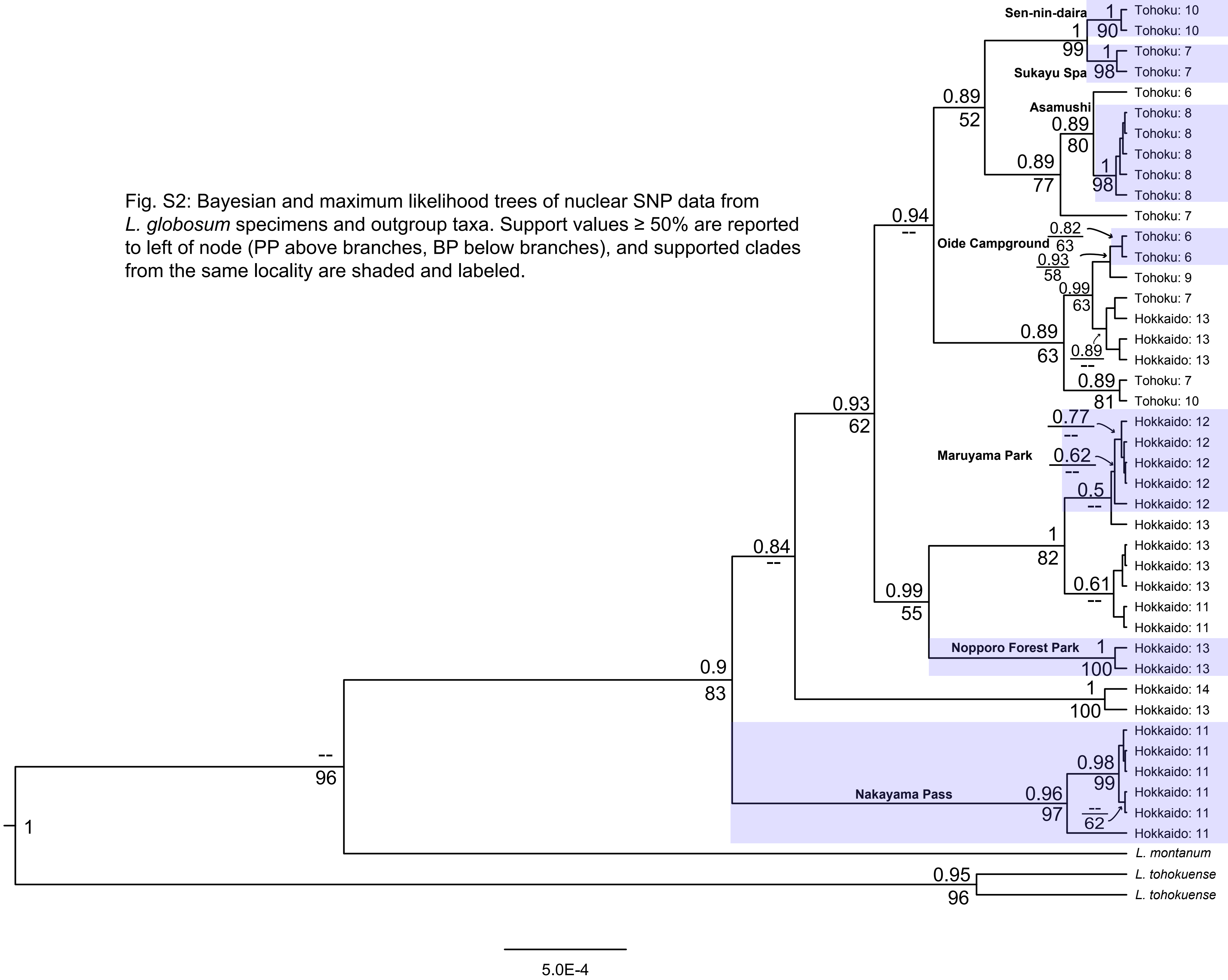

Fig. S2: Bayesian and maximum likelihood trees of nuclear SNP data from *L. globosum* specimens and outgroup taxa. Support values ≥ 50% are reported to left of node (PP above branches, BP below branches), and supported clades from the same locality are shaded and labeled.

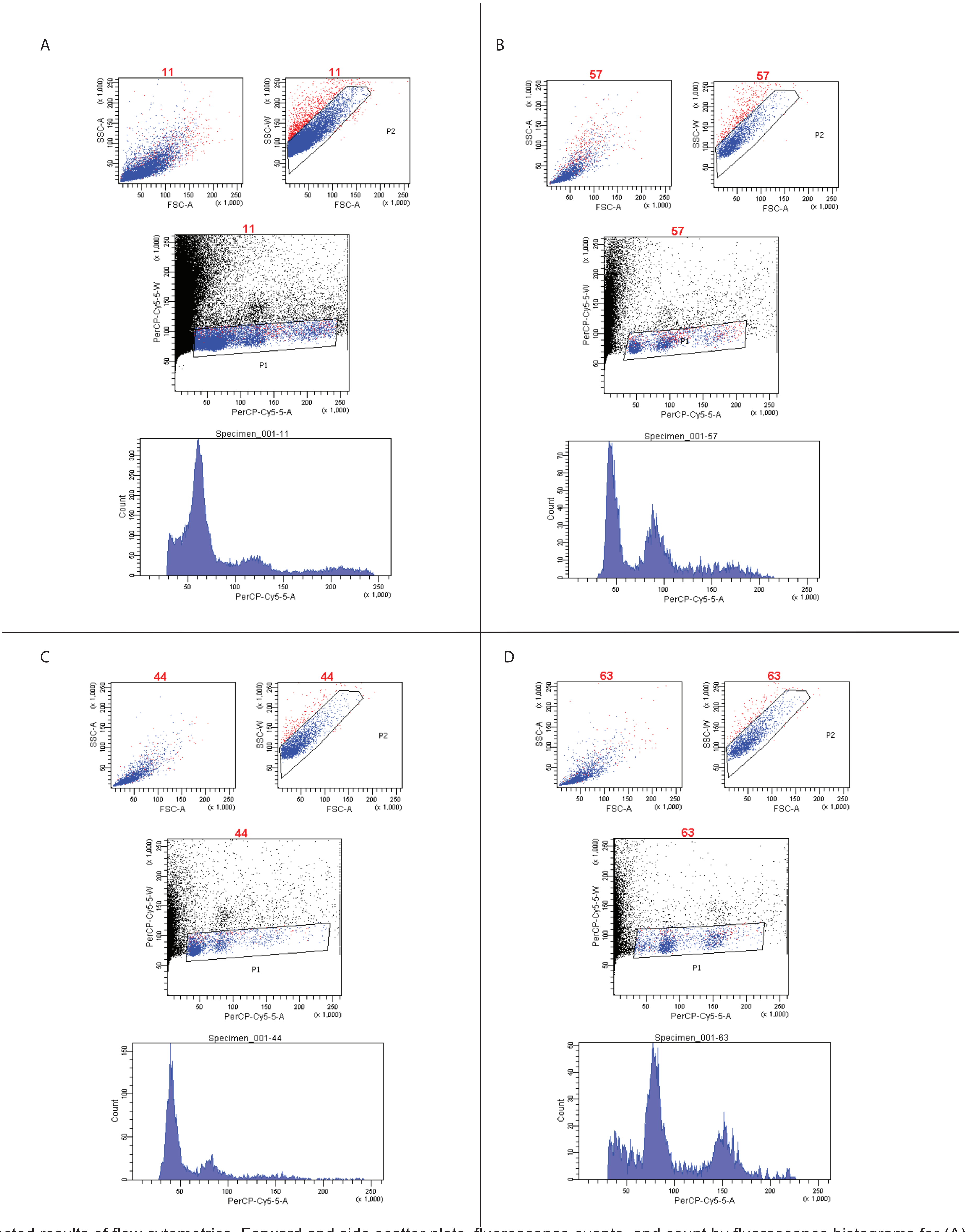

Fig. S3: Selected results of flow cytometrics. Forward and side scatter plots, fluorescence events, and count by fluorescence histograms for (A) *Leiobunum tohokuense* female #11 (used as a diploid control for comparisons), (B) *L. manubriatum* diploid female #57, from Alps: Mt. Harinoki, (C) *L. manubriatum* diploid male #44 from Alps: Mt. Harinoki, and (D) *L. manubriatum* tetraploid female #63 from Alps: Mt. Harinoki. Cell count maxima in histograms indicate cells in G1 phase; brighter peak at approximately ½ this height represents subset of mitotic cells.
